# Involvement of Cellular Prion Protein in α-Synuclein Transport in Neurons

**DOI:** 10.1007/s12035-017-0451-4

**Published:** 2017-02-22

**Authors:** Laura Urrea, Miriam Segura-Feliu, Masami Masuda-Suzukake, Arnau Hervera, Lucas Pedraz, José Manuel García Aznar, Miquel Vila, Josep Samitier, Eduard Torrents, Isidro Ferrer, Rosalina Gavín, Masato Hagesawa, José Antonio del Río

**Affiliations:** 1 Molecular and Cellular Neurobiotechnology, Institute of Bioengineering of Catalonia (IBEC), Parc Científic de Barcelona, Baldiri Reixac 15-21, E-08028 Barcelona, Spain; 2 Department of Cell Biology, Physiology and Immunology, Universitat de Barcelona, Barcelona, Spain; 3 Center for Networked Biomedical Research on Neurodegenerative Diseases (CIBERNED), Barcelona, Spain; 4 Institute of Neuroscience, University of Barcelona, Barcelona, Spain; 5 Department of Dementia and Higher Brain Function, Tokyo Metropolitan Institute of Medical Science, Setagaya-ku, Tokyo, 156-8506 Japan; 6 Bacterial infections: antimicrobial therapies. Institute of Bioengineering of Catalonia (IBEC), Parc Científic de Barcelona, Barcelona, Spain; 7 Multiscale in Mechanical and Biological Engineering (M2BE), Aragon Institute of Engineering Research, Department of Mechanical Engineering, University of Zaragoza, Zaragoza, Spain; 8 Neurodegenerative Diseases Research Group, Vall d’Hebron Research Institute-Center for Networked Biomedical Research on Neurodegenerative Diseases, Autonomous University of Barcelona, Barcelona, Spain; 9 Catalan Institution for Research and Advanced Studies (ICREA), Barcelona, Spain; 10 Nanobioengineering Group, Institute for Bioengineering of Catalonia, (IBEC), Parc Científic de Barcelona, Barcelona, Spain; 11 Department of Electronics, University of Barcelona, Martí i Franquès 1, E-08028 Barcelona, Spain; 12 Centro de Investigación Biomédica en Red en Bioingeniería, Biomateriales y Nanomedicina (CIBER-BBN), 28029 Madrid, Spain; 13 Institut de Neuropatologia, IDIBELL-Hospital Universitari de Bellvitge, Hospitalet de Llobregat, Spain; 14 Departamento de Patologia y Terapeutica Experimental, Facultad de Medicina, Universidad de Barcelona, Barcelona, Spain; 15 Centro de Investigación Biomédica en Red de Enfermedades Neurodegenerativas (CIBERNED), Barcelona, Spain

**Keywords:** Synuclein, Amyloid spreading, *Prnp*, Microfluidic devices

## Abstract

The cellular prion protein, encoded by the gene *Prnp*, has been reported to be a receptor of β-amyloid. Their interaction is mandatory for neurotoxic effects of β-amyloid oligomers. In this study, we aimed to explore whether the cellular prion protein participates in the spreading of α-synuclein. Results demonstrate that *Prnp* expression is not mandatory for α-synuclein spreading. However, although the pathological spreading of α-synuclein can take place in the absence of *Prnp*, α-synuclein expanded faster in PrP^C^-overexpressing mice. In addition, α-synuclein binds strongly on PrP^C^-expressing cells, suggesting a role in modulating the effect of α-synuclein fibrils.

**Electronic supplementary material:**

The online version of this article (doi:10.1007/s12035-017-0451-4) contains supplementary material, which is available to authorized users.

## Introduction

The cellular prion protein (PrP^C^), a glycosylphosphatidylinositol (GPI)-anchored protein, participates in several neural functions [1–3]. Relevantly, one of the most recently described functions of the protein points to PrP^C^ is as a receptor for β-amyloid (Aβ) [4]. Indeed, today it is well established that Aβ oligomers can bind with great affinity to PrP^C^ [5, 6, 4, 7, 8] and to recombinant prion protein (e.g. [9, 10]). After binding, it was also proposed, although with some controversy [9, 11–13], that this interaction plays a crucial role in neurotoxic effects of Aβ oligomers such as inhibition of long-term potentiation (LTP), neuronal cell death and memory impairment in some mouse models of Alzheimer’s disease [14, 6, 4, 15–17]. Amyloid aggregates are present in many neurodegenerative diseases, and their formation occurs in a multistep process including the misfolding of healthy soluble proteins and their association into amyloid fibrils that form cell inclusions. In fact, among other aggregates (e.g. SOD1, CEPB3, TDP-43, etc.), those of Tau and α-synuclein, characteristic of tauopathies/Alzheimer’s disease and Parkinson’s disease, respectively, showed cell-to-cell transport in healthy cells through their uptake of misfolded polymers, which can propagate and spread throughout the neural parenchyma [18–23].

α-Synuclein is a key player in the pathogenesis of synucleinopathies, including Parkinson’s disease, dementia with Lewy bodies and multiple system atrophy [24]. Transmission of synthetic α-synuclein aggregates has been demonstrated in several cellular and animal models (see [25–27] for reviews). Several groups have reported that α-synuclein shows prion-like propagation in wild-type mice [28–30]. However, the basis of the spreading process remains poorly understood although cell-to-cell transport via exocytosis has been suggested [31–33]. For this reason, PrP^C^ is proposed as an Aβ receptor (see above), and in this study, we aimed to explore whether PrP^C^ is involved in the propagation and spreading of α-synuclein. Results demonstrated that α-synuclein could propagate and spread in mice lacking or overexpressing *Prnp*, including wild-type mice. However, increased quantities of p-α-synuclein can be seen in the motor cortex of PrP^C^-overexpressing mice as compared to *Prnp* ^*+/+*^ and *Prnp* ^*0/0*^ mice. In addition, in vitro experiments also corroborated that although not required to trigger α-synuclein transport, *Prnp* overexpression enhances transported α-synuclein. In fact, α-synuclein binds strongly to *Prnp*-transfected HEK293 cells in contrast to mock-transfected ones. Moreover, the absence of the charged cluster (CC) domain of PrP^C^ impairs α-synuclein binding in transfected cells. In conclusion, our results point to a non-mandatory but relevant role of *Prnp* in α-synuclein transport.

## Material and Methods

### Mouse Strains and Genotyping

Adult male C57Bl6/129sv-*Prnp* ^0/0^ (B6129 *Prnp* ^Zrchl/Zrchl^ Zurich I) mice were purchased from the European Mouse Mutant Archive (EMMA, Monterotondo, Italy) [34]. We backcrossed to C57BL/6 J for at least 8–9 generations to obtain 6–7% of 129 microsatellites in B6.129 *Prnp* ^0/0^ and control littermates B6.129 *Prnp* ^*+/+*^ [35]. These *Prnp* ^0/0^ and *Prnp* ^+/+^ mice were used in the present study. Specific primers for *Prnp* genotyping were designed in our laboratory based on the original P3 and P10 primers as described [34]: neo: 5′-gccttctatcgccttcttgac-3′; 3′ NCnew: 5′-gctacaggtggataacccctc-3′ and P10new: 5′-cataatcagtggaacaagccc-3’ [36]. Forty cycling conditions were 45″ 95 °C; 45″ 62 °C; 1′ 72 °C, followed by a final extension at 72 °C for 5 min. *Prnp*-overexpressing mice (Tga20) were purchased from EMMA (Monterotondo, Italy). They were generated as described by Marek et al. [37] and backcrossed in our lab with B6.129 *Prnp* ^0/0^ mice for seven generations [36]. The backcrossed mice were used in the study. For Tga20 mice, the transgene was detected using primers specific to the Tg20 allele 5′-caaccgacgtgaagcattctgccta-3′ and 5′-cctgggactccttctggtaccgggtgacgc-3′ as indicated [38]. The Ethics Committee on Animal Experimentation (CEEA) of the University of Barcelona approved all procedures described in this study. All housing, breeding and procedures were performed under the guidelines and protocols #276/16 and #141/15 of CEEA.

### Preparation of Recombinant α-Synuclein Monomer and Fibrils

Mouse and human α-synuclein complementary DNAs (cDNAs) in bacterial expression plasmid pRK172 were used. α-Synuclein was expressed in *Escherichia coli* BL21 (DE3) cells and purified using boiling, Q-sepharose ion exchange chromatography and ammonium sulphate precipitation. Purified α-synuclein protein was dialyzed against 30 mM Tris–HCl, pH 7.5, and cleared using ultracentrifugation at 113,000*g* for 20 min. Protein concentration was determined with reverse phase HPLC. Proteins were loaded on an Aquapore RP-300 column (Brownlee) Perkin Elmer (Waltham, MA, USA) (equilibrated in 0.09% trifluoroacetic acid with a linear gradient of acetonitrile 0 to 50% at a flow rate of 1 ml/min). Purified mouse α-synuclein monomer (7 mg/ml) in 30 mM Tris–HCl, pH 7.5, containing 0.1% NaN_3_, was incubated at 37 °C in a shaking incubator at 200 rpm for 72 h. α-Synuclein fibrils were pelleted by spinning at 113,000*g* for 20 min and then suspended in PBS. α-Synuclein fibrils were sonicated with an ultrasonic homogenizer (VP-5S) Taitec (Nishikata, Japan) (at high power for 10 cycles (30 s on, 30 s off at 10 °C) before use. Aliquots of sonicated and non-sonicated fibrils were processed for transmission electron microscope (TEM) analysis and negative staining (Supplementary Fig. 1). In parallel, SDS-PAGE and western blot revealed the presence of a relevant 17 KDa and upper bands >35 KDa after sonication typical of protofibril preparations (Supplementary Fig. 1). To determine the concentration, fibrils were dissolved in 7 M guanidine hydrochloride and analysed with RP-HPLC as described above. Generated fibrils were tested to insure that they were endotoxin-free to avoid unwanted effects as described [39].

### Transmission Electron Microscopy Procedures

Fibril solutions were fixed to carbon-forward-coated copper supports, and negative staining was performed using a 2% PTA-based (phosphotungstic acid) stain (pH 7.4), after which samples were placed in silica-based desiccant for a minimum of 2 h. Finally, we proceeded to TEM observation using a Leica electron microscope (Wetzlar, Germany) at SCT, University of Barcelona, Barcelona, Spain (Supplementary Fig. 1).

### Stereotaxic Surgery

Five- to 6-month-old mice were anaesthetised with 50 mg/kg pentobarbital sodium and then were unilaterally injected with 5 μg (1 μg/μL solution) of recombinant mouse sonicated α-synuclein into postcommissural striatum (A-P 3.5 mm; M-L: 3.5 mm; D-V −0.9 mm from Bregma) at a rate of 0.1 μL/min. In parallel, some mice (*n* = 3) were sham-operated (Supplementary Fig. 1d). In these mice, the skull was opened and a glass pipette was placed in the abovementioned coordinates. After 10 min, the pipette was removed and the skin sutured. Sham-operated and fibril-injected mice were anaesthetised 45 days later with isofluorane and perfused with 4% buffered paraformaldehyde (pH 7.3). For immunohistochemistry, free-floating sections were rinsed in 0.1 M PBS, and endogenous peroxidase activity was blocked by incubation in 3% H_2_O_2_ and 10% methanol dissolved in 0.1 M PBS. After extensive rinsing, sections were incubated in 0.1 M PBS containing 0.2% gelatin, 10% normal serum, 0.2% glycine and 0.2% Triton X-100 for 1 h at room temperature. Afterwards, the sections were incubated for 24 h at 4 °C with the primary antibody: p-α-synuclein (p129S, clone 81A (cat. ab184674), 1:2000 diluted, Abcam, Cambridge, UK). After that, the sections were incubated with secondary biotinylated antibodies (2 h, 1:200 diluted) and streptavidin-horseradish peroxidase complex (2 h, 1:400 diluted). Peroxidase activity was revealed with 0.025% diaminobenzidine (DAB) and 0.003% hydrogen peroxide. After rinsing, the sections were mounted onto slides, dehydrated and coverslipped with Eukitt™ (Merck, Darmstadt, Germany). Samples were photodocumented using an Olympus (Hamburg, Germany) BX61 microscope equipped with a cooled digital DP72L camera. For quantification of Lewy body-like (LBL) p-α-synuclein-positive aggregates in motor cortex, equivalent sections at the level of the frontal cortex between Bregma 0 and 1 were selected (2–3 sections per mouse), and the total number of neuronal aggregates (LBL) of p-α-synuclein in layer V (300 μm *w* × 150 μm *h* box) was counted using a ×40 objective (oil immersion Zeiss, N.A. 0.85). Statistical analysis of the obtained data was performed with Bonferroni post hoc test (multiple comparison test) using GraphPad Prism 6 (Mac OsX, Graphpad). Data are presented as mean ± standard error of the mean (SEM). Differences between groups were considered statistically significant at **P < 0.01 and *P < 0.05.

### Microfluidic Devices

Two different devices were used in an optimized modification of our previous design of large dual-chamber, open neuronal co-culture and of designs reported by Taylor et al. [40]. The open microfluidic device consists of two main open chambers interconnected by 100 microchannels. The large chamber areas (9 mm × 16 mm) facilitate effective cell culture and easy handling. The small cross-section areas of microchannels (3 μm × 10 μm or 10 μm × 10 μm) restrict the crossing of cortical neuron cell bodies but permit the passage of neuronal processes. The microfluidic device was made of poly(dimethylsiloxane) (PDMS) using standard photolithography and soft lithography.

### Primary Embryonic Neuronal Cultures

E15.5 mouse embryo brains were dissected and washed in ice-cold 0.1 M phosphate-buffered saline (PBS) containing 6.5 mg/ml glucose. The meninges were removed and the cortices isolated. Tissue pieces were trypsinized for 15 min at 37 °C. After the addition of horse serum and centrifugation, cells were dissociated by trituration in 0.1 M PBS containing 0.025% DNAse with a fire-polished pipette. Dissociated cells were plated at ~10,000 cells/mm^2^ on one of the two reservoirs of microfluidic devices (A and B in Fig. 3) coated with poly-D-lysine (Sigma, Madrid, Spain). The culture medium was Neurobasal supplemented with 2 mM glutamine, 6.5 mg/ml glucose, antibiotics and B27 (Invitrogen-Life Technologies, Barcelona, Spain). After 72 h, 5 μM AraC (cytosine β-D-arabinofuranoside hydrochloride, Sigma) was added for 48 h to inhibit the growth of dividing non-neuronal cells. For characterization, cultures were immunostained using anti-glial fibrillary acidic protein (GFAP, cat. z0334; 1:500, Sigma), basic helix-loop-helix (bHLH) transcription factor olig2 (cat. AB9610, 1:200; Abcam) and class III β-tubulin (TUJ1, cat. 801201, 1:3000; Biolegend, CA, USA). Cultures contained up to 90% neurons (TUJ1-positive) and were used after 5–7 days in vitro. In parallel, some cultures were stained with Fluo4-AM (1 mg/ml, F14201, Invitrogen-Life Technologies) to confirm axonal interconnection between reservoirs prior to their processing.

### Plasmids and Construction of PrP^C^-Deleted Forms

Mouse PrP^C^-encoding plasmid (pcDNA 3.1 backbone) and PrP^C^-IRES-GFP were provided by D. A. Harris (Boston University School of Medicine, Boston, MA, USA) and PrP^C^-truncated form ΔF35 by A. Aguzzi (University Hospital Zürich, Institute for Neuropathology, Switzerland). To generate deletion constructs ΔCD, ΔCC, ΔHR and ΔCR, the PrP^C^-encoding plasmid was used as a template for inverse PCRs, and the inserts obtained were fused. Briefly, a primer set was designed for each construct in order to amplify the entire plasmid, except for the region of *Prnp* to be deleted, i.e. regions 95–133, 95–110, 112–133 and 105–125 for ΔCD, ΔCC, ΔHR and ΔCR, respectively. Primers (Ecogen) were as follows: CD (F: 5′-AGCAGGCCCATGATCCATTTTG-3′, R: 5′-ATGGGTACCCCCTCCTTGGC-3′); CC (F: 5′-GTGGCAGGGGCTGCGGCAG-3′, R: 5′-ATGGGTACCCCCTCCTTGGCC-3′); HR (F: 5′-AGCAGGCCCATGATCCATTTTG-3′, R: 5′-TGCCACATGCTTGAGGTTGG-3′); CR (F: 5′-TACATGCTGGGGAGCGCC-3′, R: 5′-TTTTGGTTTGCTGGGCTTGTTC-3′). After amplification (Accuprime Taq Polymerase™, Invitrogen), the blunt ends of the amplimers were phosphorylated using the T4 kinase reaction (Invitrogen) and then religated (Fast-Link Ligase™, Epicentre Biotech.). An aliquot of each ligation reaction was electroporated into *Escherichia coli* DH5α, and transformants were selected for ampicillin resistance. Twenty-five candidates were selected and screened with sequence analysis (Terminator Big Dye™ v3.1, Applied Biosystems).

### Cell Culture and Transfection

HEK293 cells (ATCC CRL-1573™, American Type Culture Collection, MD, USA) were maintained in Dulbecco’s modified eagle medium (DMEM, Invitrogen-Life Technologies), 10% foetal bovine serum (FBS, Invitrogen-Life Technologies) and 1% penicillin/streptomycin (Invitrogen-Life Technologies) in 75 cm^2^ culture bottles in a 5% CO_2_ atmosphere at 37 °C. One day before transfection, cells were cultured in DMEM supplemented with 10% FBS and without antibiotics, on poly-D-lysine (Sigma) coated plates. Transfection was performed using Lipofectamine Plus (Invitrogen-Life Technologies), according to the manufacturer’s instructions as indicated [41].

### Caspase-3 Activity

Twenty-four hours after transfection, cells were scraped and lysed for 20 min on ice in cold lysis buffer (50 mM Hepes pH 7.5, 150 mM NaCl, 1.5 mM MgCl_2_, 1 mM EGTA pH 8, 10% glycerol, 1% Triton X-100) containing 1x protease inhibitor cocktail and phosphatase inhibitors. Lysates were centrifuged at 12000*g* for 5 min at 4 °C and supernatants were collected. Protein concentration was determined using a BCA protein assay kit (Pierce). The caspase-3 activity assay, with Ac-Asp-Glu-Val-Asp-7-amino-4-trifluoromethylcoumarin (Ac-DEVD-AFC, Sigma) as a substrate, was performed as previously described [42].

### Immunocytochemical Procedures

One day before transfection, counted HEK293 cells were seeded onto poly-D-lysine (0.01 μg/μl) coated glass coverslips (12 mm Ø). Three days post-transfection, mouse α-synuclein (1 μg/ml medium) was added to the culture media. Treatment with α-synuclein was maintained for 3 days. Cells were fixed in 4% buffered paraformaldehyde (Sigma) and then permeabilized with 0.1% Triton X-100 (Sigma) in 0.1 M PBS. A similar fixation procedure was used for microfluidic devices (see [43] for details). After fixation, and extensive rinsing with 0.1 M PBS, cultures were blocked with 10% FBS in 0.1 M PBS prior to incubation with primary antibodies. Neurons were identified using a class III β-tubulin antibody (1:3000 diluted; Biolegend). PrP^C^ was detected using anti-mouse 6H4 (1:500, Prionics; Schlieren, Switzerland), which recognizes the sequence DYEDRYYRE of the prion protein (human PrP^C^: aa 144–152), α-synuclein (1:500 diluted; Cell Signalling) and p-α-synuclein (mouse and human) (p129S/81A; 1:400 diluted; Abcam). After incubation with primary antibodies, cells were incubated with the pertinent Alexa Fluor-tagged secondary antibodies (Alexa-488 goat anti-mouse or Alexa-568 goat anti-rabbit) (Invitrogen-Life Technologies). Finally, cells were stained with 0.1 μM DAPI (Sigma) diluted in 0.1 M PBS, mounted on Mowiol™ (Calbiochem, San Diego, CA, USA) and viewed using an Olympus BX61 fluorescence microscope.

### Western Immunoblot

Samples were homogenized in 10% wt/vol of 50 mM Tris–HCl, pH 7.4/150 mM NaCl/0.5% Triton X-100/0.5% Nonidet P-40, and a mixture of protease inhibitor cocktail (Roche, Basel, Switzerland) and phosphatase inhibitors (10 mM tetra-sodium pyrophosphate, 200 μM sodium orthovanadate and 10 mM sodium fluoride). After this, samples were centrifuged at 15,000*g* for 20 min at 4 °C. The resulting supernatant was normalized for protein content using BCA kit (Thermo Scientific Pierce, Paisley, UK). Cell extracts were boiled at 100 °C for 10 min, followed by 6% SDS electrophoresis and were then electrotransferred to nitrocellulose membranes for 1 h at 4 °C. Membranes were then blocked with 5% fat milk in 0.1 M Tris-buffered saline (pH 7.4) for 1 h and incubated overnight in 0.5% blocking solution containing primary antibodies. After incubation with peroxidase-tagged secondary antibodies (1:2000 diluted, Sigma), membranes were revealed with an ECL-plus chemiluminescence western blot kit (Amershan-Pharmacia Biotech, Piscataway, NJ, USA). In some experiments, peroxidase activity was revealed using a high sensitivity ECL-chemiluminescence kit (QuantaRed™, Thermo Scientific). In our experiments, each nitrocellulose membrane was used to detect α-synuclein (1:1000; Cell Signalling), p-α-synuclein (1:1000, p129S/81A; Abcam), β-actin (1:20,000; Sigma), β-tubulin (1:10,000; Sigma) and class III β-tubulin antibody (1:5000; Biolegend).

### Densitometry and Statistical Processing

For quantification, developed films were scanned at 2400 × 2400 dpi (i800 MICROTEK high quality film scanner), and the densitometric analysis was performed using the Quantity One Image Software Analysis (Biorad, Barcelona, Spain). Statistical analysis of the obtained data was performed with Bonferroni post hoc test (Multiple comparison test) using GraphPad Prism 6 (Mac OsX, Grahpad). Data are presented as mean ± SEM. Differences between groups were considered statistically significant at *P < 0.05.

## Results

### α-Synuclein Transport in *Prnp*^*+/+*^, *Prnp*^0/0^ and Tga20 Mice

**Fig. 1:**
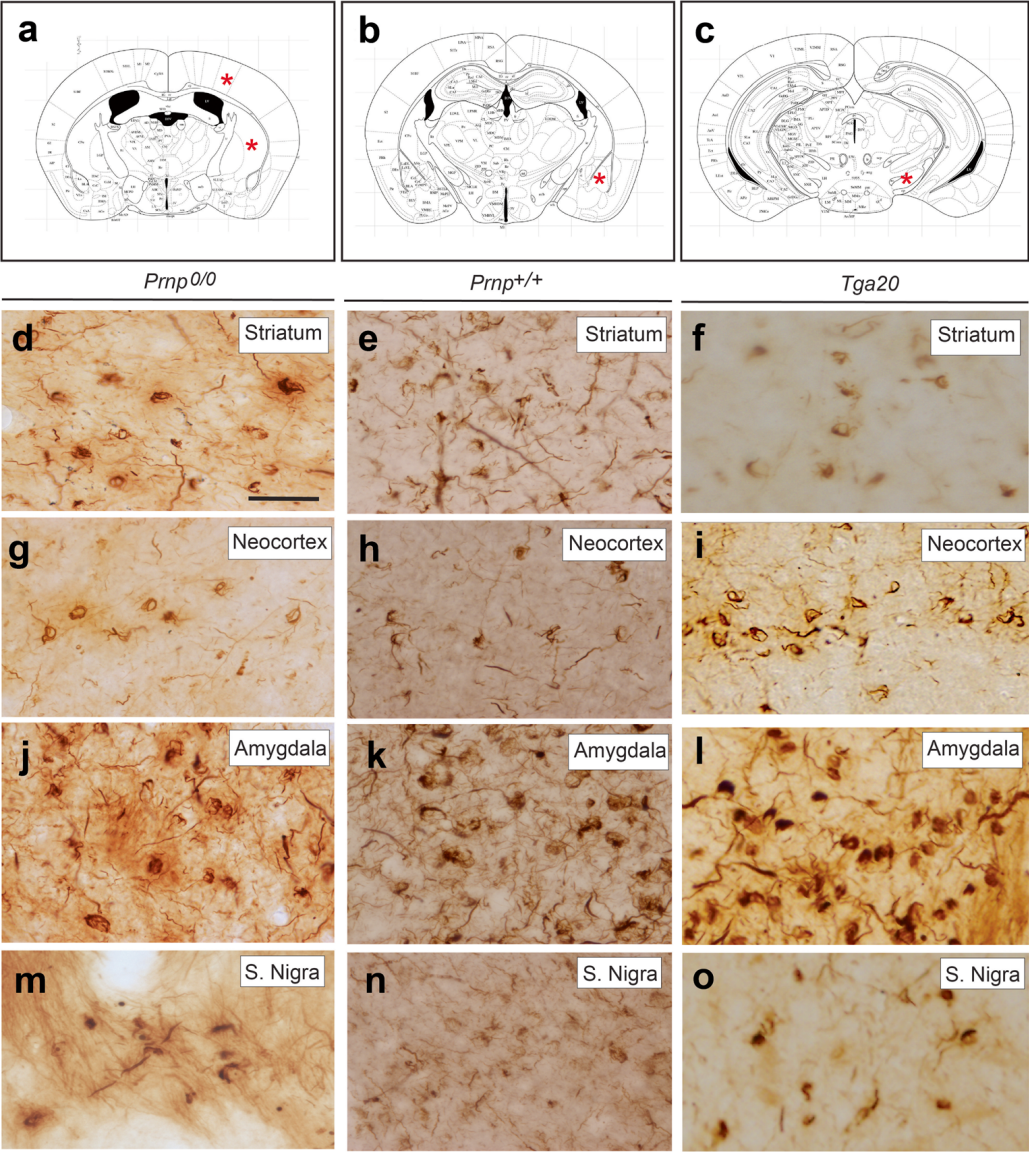
p-α-Synuclein pathology in the telencephalon of *Prnp* ^0/0^, *Prnp* ^+/+^ and Tga20 mice injected with mouse α-synuclein fibrils in the postcommissural striatum. **a-c** Schemes illustrating the location of p-α-synuclein deposits (*asterisks*) shown in panels (**d**–**o**). **d-o** High power photomicrographs showing p-α-synuclein labelling in the striatum (**d**–**f**); neocortical layer V (**g**–**i**); amygdala (**j**–**l**) and S. nigra (**m**–**o**) of *Prnp* ^0/0^ (**d**, **g**, **j**, **m**), *Prnp* ^+/+^ (**e**, **h**, **k**, **n**) and Tga20 (**f**, **i**, **l**, **o**) mice. Note the relevant accumulation of p-α-synuclein labelling in intracellular deposits of retrograde-labelled projecting neurons. *Scale bar*: **d** = 100 μm pertains to **e**–**o**

Adult *Prnp* ^*+/+*^, of recombinant endotoxin-free mouse α-synuclein (Fig. 1) in the postcommissural striatum, and the presence of phosphorylated α-synuclein (p-α-synuclein, p129S/81A–positive), were analysed 45 days later. Both sham- and non-operated mice presented the described pattern of p-α-synuclein in the adult mouse telencephalon (Supplementary Fig. 1, [44]). The antibody p129S/81A showed minor cross-reactivity with phosphorylated Neurofilament L. Indeed, several telencephalic regions showed background staining including the cingulate and parietal neocortex, hippocampus, striatum and some telencephalic axonal tracts (e.g. mamillothalamic tract) (Supplementary Fig. 1) [44]. However, aggregated forms of p-α-synuclein such as Lewy body-like (LBL) or Lewy neurite-like (LNL) forms were never observed after immunohistochemistry using the p129S/81A antibody in the telencephalon of non-operated and sham-operated mice, only appearing in the telecephalon after α-synuclein fibril injections (Supplementary Fig. 1). High magnification of LBL observed in the neocortex and amygdala 45 days after mouse α-synuclein striatal injections can be seen in Supplementary Fig. 1f, g. As observed, these aggregates were clearly identifiable over the background even using Ni-DAB development.

**Fig. 2:**
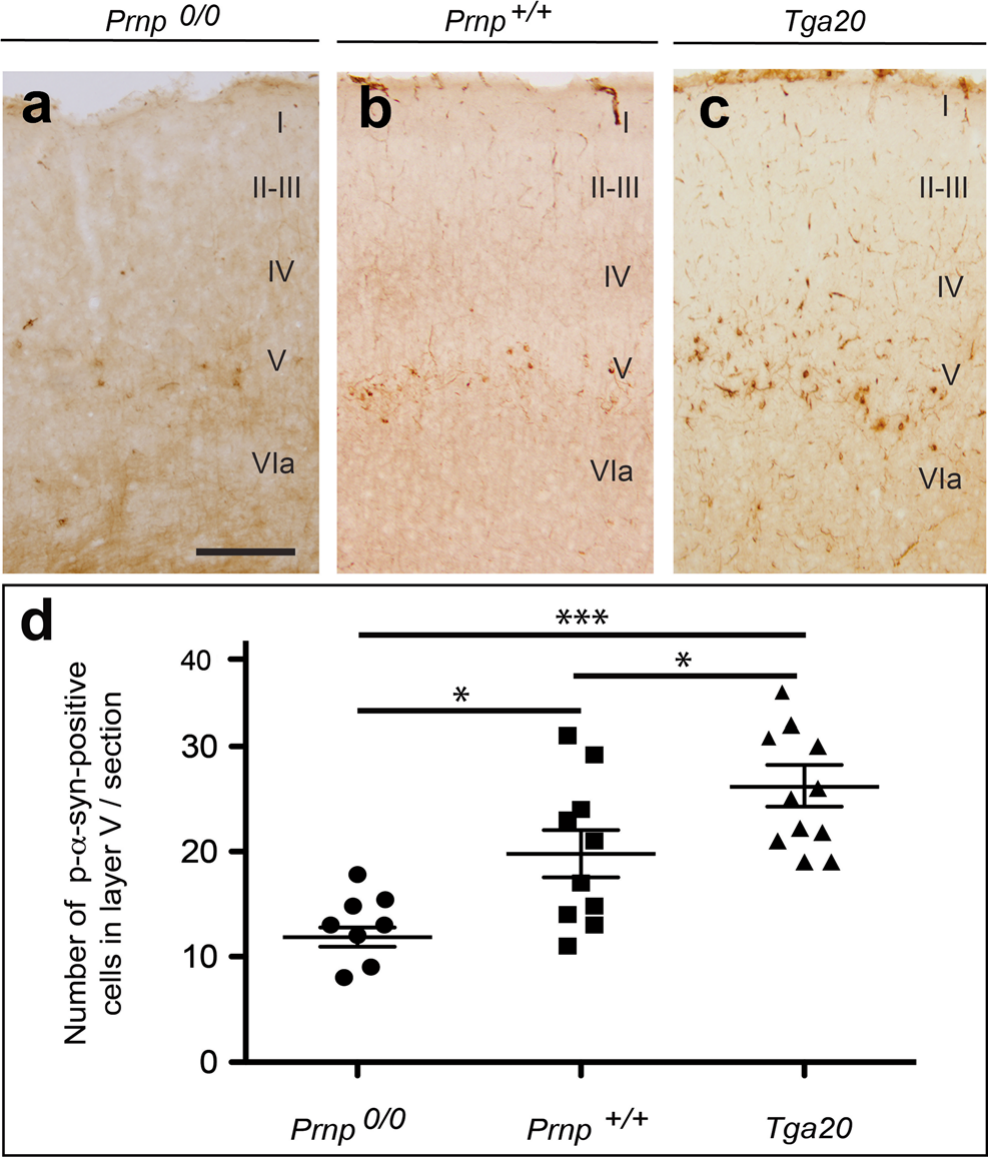
Increased α-synuclein labelling as LBL aggregates in the neocortex in Tga20 mice. **a**–**c** Examples of p-α-synuclein aggregates in the motor cortex of *Prnp* ^*0/0*^, *Prnp* ^+/+^ and Tga20 mice injected with mouse α-synuclein fibrils in the postcommissural striatum. Note the relevant accumulation of p-α-synuclein labelling in intracellular deposits of retrograde-labelled neurons in the cortical layer V of Tga20 mice. The LBL and LNL aggregates can be clearly seen over the pale background in the cortex. **d** Graph illustrating the quantification of the neuronal aggregates in the different genotypes. Each count represents one section. (Mice number *Prnp* ^*+/+*^ (*n* = 5); *Prnp* ^*0/0*^ (*n* = 4) and Tga20 (*n* = 6)). In addition, the mean ± SEM is also plotted. *P < 0.05 and ***P < 0.01, ANOVA Bonferroni post hoc test. *Scale bar*: **a** = 100 μm pertains to **b** and **c**

As indicated, the mice were processed after 45 days post-injection (*Prnp* ^*+/+*^ (*n* = 6); *Prnp* ^0/0^ (*n* = 6) and Tga20 (*n* = 7)) in order to determine whether the regional distribution of p-α-synuclein was similar in the different *Prnp* genotypes (Figs. 1 and 2, Supplementary Fig. 1). Injection of mouse α-synuclein sonicated fibrils into postcommissural striatum induced p-α-synuclein pathology bilaterally throughout the brain, including striatum, amygdala, stria terminalis, substantia nigra and neocortex (Figs. 1 and 2, Supplementary Fig. 1). The anatomical and cellular distribution of p-α-synuclein aggregates was observed in *Prnp* ^*+/+*^, *Prnp* ^0/0^ and Tga20 mice, as previously reported for wild-type mice in another study using similar α-synuclein fibrils and protocols [30]. At the cellular level, p-α-synuclein was mainly located in neurites and the neuronal perikaryon (as thick LBL or LNL aggregates) (Figs. 1 and 2, Supplementary Fig. 1e, f). At these post-inoculation times, p-α-synuclein labelling was observed bilaterally in the striatum, substantia nigra, amygdala, and entorhinal cortex and, ipsilaterally, in the motor cortex. However, although α-synuclein pathology was observed in the absence of *Prnp*, histological examination and quantitative analysis of genotypes determined increased p-α-synuclein staining as LBL in the ipsilateral motor cortex of Tga20 mice compared to *Prnp* ^0/0^ and *Prnp* ^*+/+*^ (*Prnp* ^0/0^ = 11.86 ± 0.91; *Prnp* ^*+/+*^ = 19.80 ± 2.25; Tga20 = 27.56 ± 2.19 (mean ± SEM). *Prnp* ^0/0^ vs *Prnp* ^*+/+*^, mean diff −7.94, *t* = 2.669, 95% CI of diff = −15.63 to −0.2592. *Prnp* ^0/0^ vs Tga20, mean diff −15.70, *t* = 5.159, 95% CI of diff = −23.56 to −7.84. *Prnp* ^*+/+*^ vs Tga20, mean diff −7.756, *t* = 2.795, 95% CI of diff = −14.92 to −0.59; ANOVA Bonferroni multiple comparison test) (Fig. 2). These data strongly suggest that although non-mandatory, *Prnp* overexpression enhances the regional transport of p-α-synuclein pathology in living mice.

### Development of Microfluidic Devices to Monitor α-Synuclein Fibril Transport

**Fig. 3:**
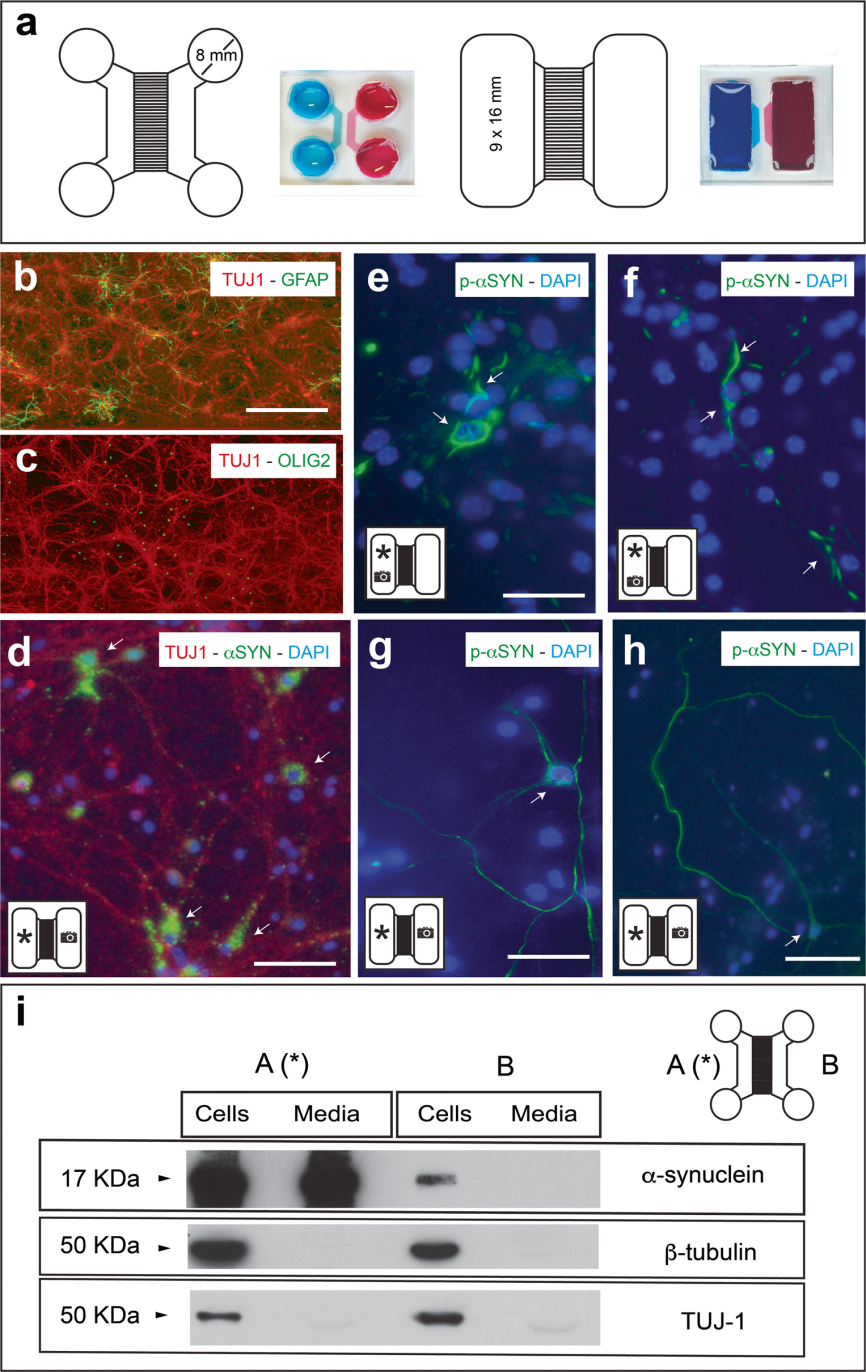
Analysis of α-synuclein transport using microfluidic devices. **a** 2D representation of the two PDMS devices used in the present study. **b**, **c** Double immunofluorescence photomicrographs illustrating TUJ1/GFAP (**b**) or TUJ1/olig2 (**c**) staining in primary cultured neurons in the devices. **d** Primary cortical cultures of the *Prnp* ^+/+^ were maintained in the devices for 7 days. Then, mouse α-synuclein fibrils were added to A reservoir (*asterisk*), and their transport to B reservoir was analysed with immunocytochemistry (**d**–**h**) and western blot (**i**). **d** Examples of double-labelled neurons (TUJ1/α-synuclein) in B reservoir (indicated with *camera icon*) showing α-synuclein labelling (*arrows*). **e**, **f** Examples of p-α-synuclein-labelled neurons and neurites (*arrows*) in A reservoir (indicated with *camera icon*). **g**, **h** Examples of p-α-synuclein-labelled neurons and axons (*arrows*) in B reservoir (indicated with *camera icon*). **i** Western blot showing the presence of α-synuclein (17 kDa band) in both cellular extracts (A and B) and the absence in the culture medium of B in contrast to A reservoir, avoiding passive fluid transport. Membranes were reblotted with an antibody against β-tubulin or TUJ1 for standardization and characterization. *Scale bar*: **b**, **d**, **e**, **g** and **h** = 40 μm

α-Synuclein intracellular transport has been studied in vitro using commercial microfluidic devices (e.g. [45–48]). Our laboratory has developed two microfluidic designs based on previously published devices of our group (Fig. 3) [43]. Two different types of microgrooves (both 1 mm length) were generated for this study: 100 microgrooves of 3 μm (high) × 10 μm (wide) sections and 100 microgrooves of 10 (high) × 10 μm (wide) sections (Fig. 3a). Results in protein transport were similar for the two devices since microfluidic pressures were controlled to avoid non-specific non-neuronal transport (see below). As noted, α-synuclein transport in vitro can be done both anterogradely and retrogradely [45–47]. Thus, we cultured *Prnp* ^*+/+*^ embryonic (E15.5) cortical neurons for 5–7 days in our devices to ensure crossing of axons between A and B. Under these conditions, around ≈85% of the devices showed large numbers of crossing axons after Fluo4-AM staining between reservoirs (Supplementary Fig. 2a, b). In addition, immunohistochemical analysis demonstrated that a large number of TUJ1-positive neurons with fewer astrocytes (glial fibrillary acidic protein (GFAP)-positive) and oligodendrocytes (olig2-positive) were observed in the devices (Fig. 3b, c). Recombinant (1 μg/ml) mouse (Fig. 3) or human (Supplementary Fig. 3) α-synuclein fibrils were added to one of the reservoirs (mainly in A; (A *) in Fig. 3) following the protocol of [46] with different volumes of medium between reservoirs (B > A; 100 μl), and their transport (A ⇒ B) were analysed 5 days later with immunocytochemistry (Fig. 3d–h, Supplementary Fig. 3) or western blot (Fig. 3i and Supplementary Figs. 2 and 4).

First, we aimed to determine endogenous α-synuclein and p-α-synuclein labelling in our devices. At these stages, a very low α-synuclein immunostaining was observed in untreated devices in both A and B reservoirs. In addition, after using the p-α-synuclein (p129S/81A) antibody, no labelling was observed in the absence of α-synuclein fibril cultures. However, after α-synuclein fibril treatment in A (Fig. 3d), immunoreacted cultures showed the presence of relevant α-synuclein staining in the perikaryon, neurites and axons of cultured TUJ1-positive neurons in B (Fig. 3d). In addition, relevant p-α-synuclein labelling was observed in morphologically identified neurons located in both reservoirs (A and B) (Fig. 3e–h**)**. More relevantly, p-α-synuclein aggregates (after mouse or human fibril treatment) were detected in identified axons and in the cytoplasm of cultured neurons in A as well in B (Fig. 3e–h, Supplementary Fig. 3b–g). In addition, to avoid the presence of passive transport of α-synuclein, we developed the detection of the protein in cell extracts and media of both reservoirs using the QuantaRed™-enhanced chemifluorescent HRP substrate (Thermo Fisher, cat. 15159) (Fig. 3i and Supplementary Figs. 2 and 4). After mouse α-synuclein fibril incubation in A, results revealed the presence of α-synuclein in both cellular extracts (A and B), with the absence in the culture media of B in contrast to the media of A reservoir, indicating the absence of passive fluidic flux between A ⇒ B (Fig. 3i and Supplementary Figs. 2 and 4) [43, 46]. In our devices, although different α-synuclein bands could be detected in A, the low molecular weight of α-synuclein (≈17 kDa) was mainly detected in B, as also recently reported by [47](Fig. 3, Supplementary Figs. 2 and 4). α-Synuclein transport was always observed in those devices showing large connectivity between A and B reservoirs (Fig. 3, Supplementary Figs. 3 and 4). However, in those displaying few axon numbers crossing between reservoirs (Supplementary Fig. 3), α-synuclein was almost absent in B (cell and media) and was only detected in the cellular and media extracts of A. This reinforced the notion of a specific axonal transport of α-synuclein fibrils between neurons displaying low endogenous levels of α-synuclein at these time points in culture (Supplementary Fig. 2). Thus, these devices are well suited to monitor cellular transport of mouse or human α-synuclein fibrils.

### In Vitro Transport of α-Synuclein under *Prnp* Dosage

**Fig. 4:**
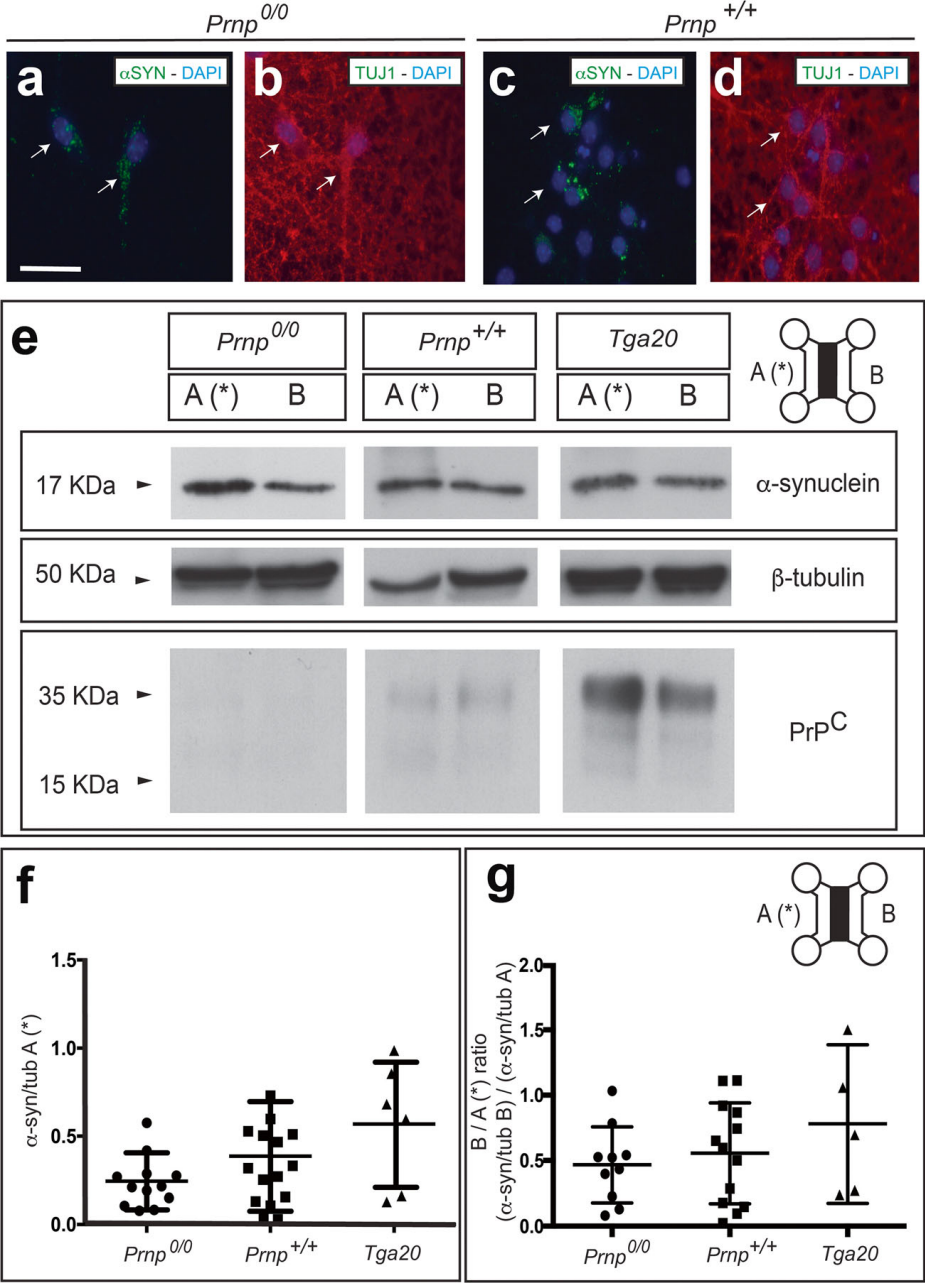
Determination of α-synuclein transport in neuronal cultures from mouse embryos carrying differing *Prnp* dosages. Recombinant mouse α-synuclein protofibrils were added to A reservoir (*asterisk*), and its binding to neurons in A reservoir as well as their transport towards B were analysed by western blot in cellular extracts. **a**–**d** Examples of double-labelled *Prnp* ^*0/0*^ (**a**, **b**) and *Prnp* ^*+/+*^ (**c**, **d**) neurons (TUJ1/α-synuclein) in B reservoir showing discrete cytoplasmatic α-synuclein labelling (*arrows*). **e** Examples of western blot determination of one device for each *Prnp* genotype for α-synuclein. Anti-β-tubulin and anti-PrP^C^ monoclonal antibodies were used for standardization and genotype characterization. Notice that PrP^C^ was present in WT and Tga20 cells at the time of treatment. PrP^C^ is downregulated shortly after plating neurons, and their levels increased over time in culture (see also [68] for details). **f**–**g** Densitometric analysis (see the “Material and Methods” section for details) were performed, and quantification was represented as the ratio between α-synuclein/β-tubulin detected in A reservoir after protofibril treatment (**b**) and ratio between α-synuclein/β-tubulin detected in B vs A reservoirs (**c**). Results for each device are represented by a single plot in the scatter plot. In addition, mean ± SEM is also plotted

As indicated above, PrP^C^ has been described as a receptor for β-amyloid [4, 49]. Although the participation of PrP^C^ in the intercellular β-amyloid transport in microfluidic devices remains elusive, its expression seems to be needed to trigger β-amyloid-mediated effects in treated neurons (see the “Introduction” section for references). Primary cortical cultures from *Prnp* ^*+/+*^, *Prnp* ^0/0^ and Tga20 embryos were treated with exogenous recombinant mouse α-synuclein fibrils, and both the binding in treated cells and their transport was analysed with western blot or immunocytochemistry (Fig. 4). First, we checked the percentage of devices without the α-synuclein transport. Results indicated that 14.28% (*Prnp* ^0/0^), 22.22% (*Prnp* ^*+/+*^) and 16.66% (Tga20) of the cultures showed no α-synuclein transport in our microfluidic devices. In fact, these cultures displayed very few axons crossing between reservoirs as determined by Fluo4-AM staining (see example in Supplementary Fig. 2a). Second, we analysed the presence of exogenous α-synuclein in neurons in B reservoir after treatment in A. We were able to determine the presence of α-synuclein in neurons, irrespective of the *Prnp* genotype (Fig. 4a–d). Since neuronal presence of α-synuclein fibrils cannot be ascertained in a quantitative basis by simple fluorescence analysis, we processed parallel samples for western blot. Indeed, after protofibril treatment in A, western blots revealed increased α-synuclein/tubulin ratio in Tga20 than in WT and *Prnp* ^0/0^ cultured neurons in A suggesting a tendency of higher protofibril binding in presence of larger PrP^C^ amounts (*Prnp* ^0/0^ *=* 0.344 ± 0.07; *Prnp* ^*+/+*^ = 0.386 ± 0.08, Tga20 = 0.56 ± 0.14 (mean ± SEM)) (Fig. 4b). Next, western blot results corroborated that α-synuclein protofibrils could be transported to neurons from A to B irrespective of the *Prnp* genotype (Fig. 4). However, although not statistically significant (ANOVA Bonferroni post hoc test), values for transported α-synuclein were slightly higher in Tga20-derived primary neuronal cultures as compared to wild-type and *Prnp* ^0/0^ (Fig. 4c) (*Prnp* ^0/0^ *=* 0.467 ± 0.09; *Prnp* ^*+/+*^ = 0.5561 ± 0.106, Tga20 = 0.7801 ± 0.206 (mean ± SEM)).

### Increased Binding of α-Synuclein in *Prnp*-Transfected Cells

**Fig. 5:**
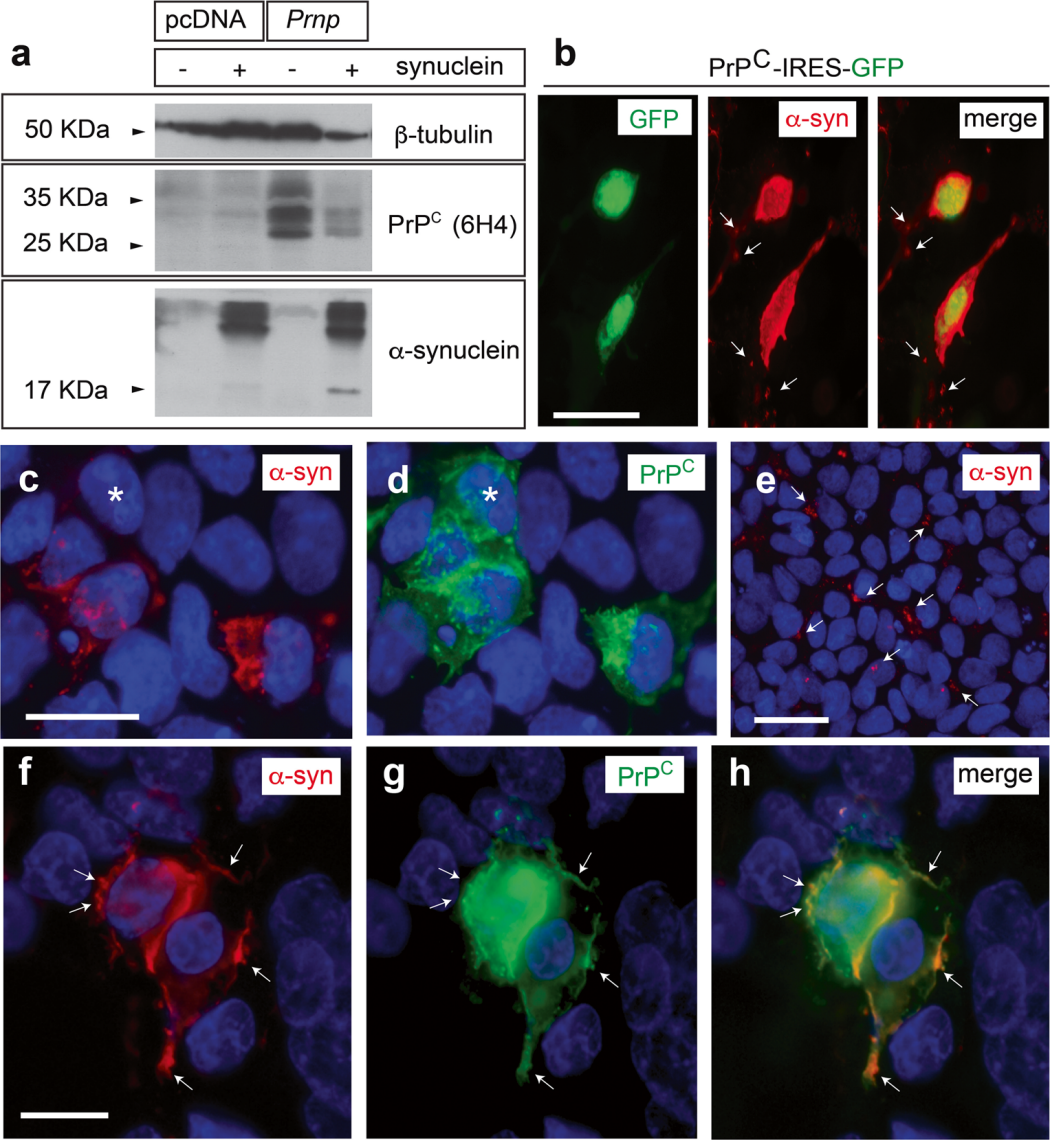
Increased binding of α-synuclein in *Prnp*-transfected HEK293 cells. **a** Western blot shows increased labelling of the 17 KDa α-synuclein band in HEK293 cells transfected with mouse *Prnp*-encoding plasmid in contrast to mock-transfected cells. Anti-β-tubulin monoclonal antibody was used for standardization, and anti-PrP^C^ antibody was used to check PrP^C^ overexpression after transfection. Notice that HEK293 cells showed a very low endogenous PrP^C^ expression. The upper bands observed after protofibril treatment corresponded to non-monomeric forms of α-synuclein. **b** Examples of double-labelled GFP/α-synuclein HEK293 cells after transfection of PrP^C^-IRES-GFP. Note the presence of the relevant labelling in the two transfected cells in comparison to the disperse α-synuclein labelling in non-transfected cells (*arrows*). **c**–**e** Fluorescence photomicrographs showing examples of double-labelled cells PrP^C^ (**d**) overexpressing cells and α**-**synuclein (**c**). HEK293 cells were transfected with *Prnp*-encoding plasmid (**c**, **d**) or mock-transfected cells (**e**). *Arrows* in **c** and **d** point to double-labelled cells, and the *asterisk* in **d** labels a PrP^C^-positive/α-synuclein-negative HEK293 cell. *Arrows* in **e** point to α-synuclein labelling in mock-transfected cells. **f**–**h** High magnification photomicrograph illustrating the distribution of α-synuclein (**f**) in PrP^C^-transfected HEK293 cells (**g**). Notice the relevant colocalization in several domains of the transfected cell including the plasma membrane (arrows in **g** and **h**). *Scale bars*: **b**, **e** *=* 25 μm. **c**, **f** = 25 μm belongs to **d**; **g** and **h**, respectively

**Fig. 6:**
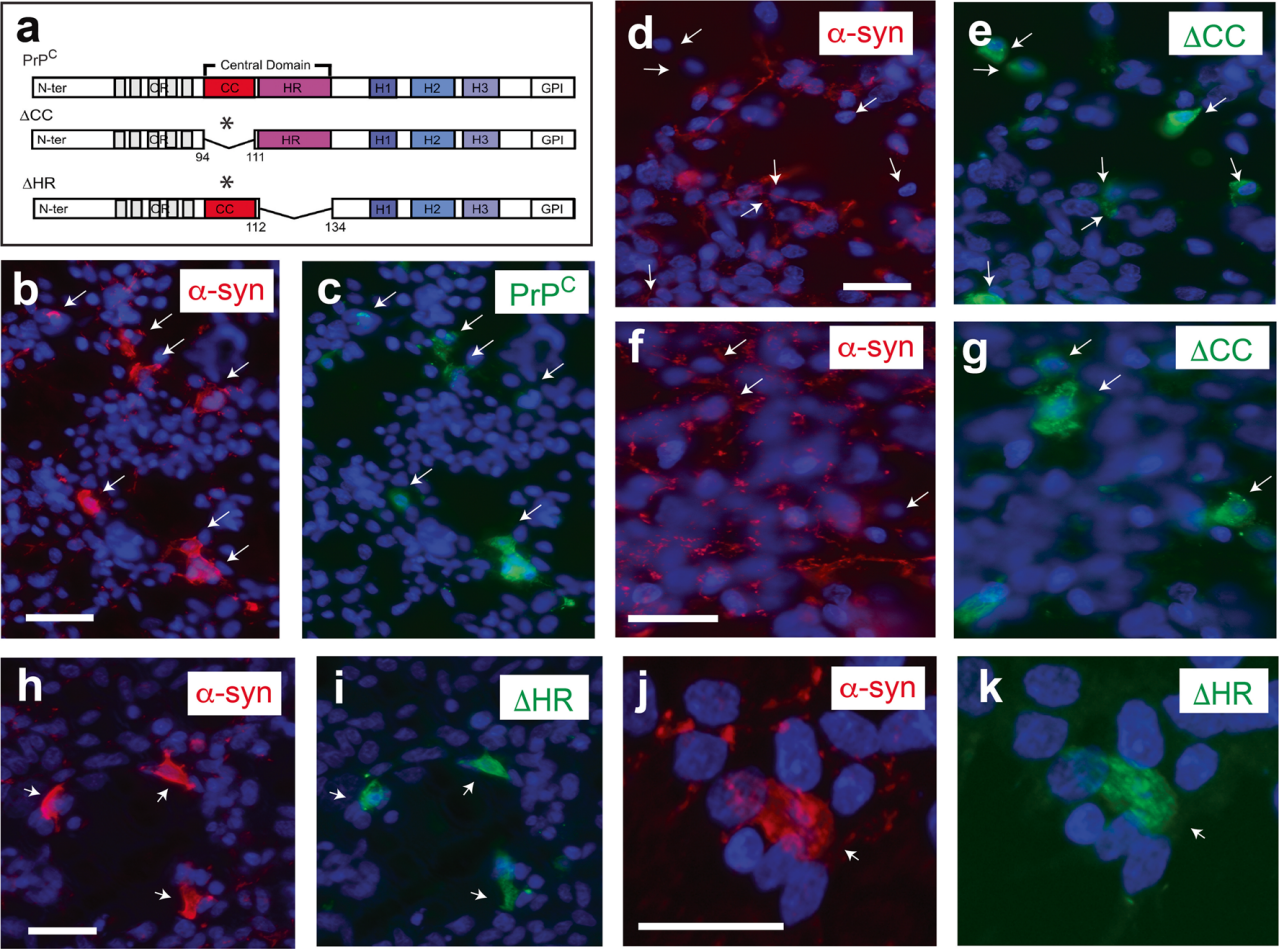
**a** Scheme of the ΔCC, ΔHR maps and the full**-**length PrP^C^. **b**, **c** Fluorescence photomicrographs showing examples of double-labelled PrP^C^/α**-**synuclein cells (*arrows*). **d**, **e** Fluorescence photomicrographs showing examples of double-labelled cultures using the 6H4 PrP^C^ and α-synuclein antibodies. Note the absence of double-labelled cells in these examples (*arrows*) and the background staining of α-synuclein labelling in non-transfected cells. **h**–**k** Fluorescence photomicrographs showing examples of double-labelled ΔHR/α-synuclein cells (*arrows*) after treatment over the labelling of non-transfected cells. *Scale bar*: **b**, **d**, **f**, **h** and **j** = 40 μm pertains to **c** and **e**, respectively

Various possibilities have been proposed regarding α-synuclein interaction with plasma membrane [50–52, 48]. Thus, due to the in vitro and in vivo observations, we aimed to determine whether PrP^C^ overexpression enhanced α-synuclein interaction with cells (Figs. 5 and 6 and Supplementary Fig. 5). We increased PrP^C^ expression in a cellular system with very low PrP^C^ expression, low endogenous α-synuclein binding properties [48] and lack of expression of other α-synuclein binding proteins (LAG3, neurexin 1β or APLP1) (HEK293 cells, Fig. 5) to analyse the binding of α-synuclein mouse protofibrils. First, HEK293 cells were transfected with PrP^C^-IRES-GFP, incubated with sonicated mouse α-synuclein protofibrils (1 μg/ml) and processed for α-synuclein immunolabelling (Fig. 5b). Results revealed that most GFP-positive HEK203 cells were labelled with the α-synuclein antibody (Fig. 5b). In a second set of experiments, HEK293 cells were transfected either with full-length *Prnp* (pcDNA-*Prnp*) or mock (pcDNA) plasmids and then incubated 24 h later with mouse α-synuclein fibrils; their cellular binding was analysed using western blot and immunocytochemistry for PrP^C^ and α-synuclein (Fig. 5a, c–h). Blots indicated the presence of the 17 kDa α-synuclein band only in protein extracts of *Prnp*-transfected cells after α-synuclein treatment (Fig. 5a). This increased binding was also corroborated by immunocytochemistry in which α-synuclein labelling was prominent in identified double-labelled *Prnp*-expressing cells in contrast to non-expressing HEK293 cells (Fig. 5c–h). Fluorescence microscopy observation developed in 314 (PrP^C^-positive) identified cells from three different experiments demonstrated that 91.08% of the analysed cells with relevant α-synuclein labelling were also positive for PrP^C^ (Fig. 5c, d). In contrast, a discrete puncta-like labelling of α-synuclein was observed randomly distributed over mock-transfected HEK293 cells with negligible levels of PrP^C^ (Fig. 5e). At high magnification, although not exclusive, we determined a strong co-localization of the α-synuclein and PrP^C^ labelling in discrete membrane regions of transfected cells, suggesting relevant cellular binding of protofibrils in regions with high PrP^C^ presence (Fig. 5f–h).

### Involvement of the Charged Cluster Domain of PrP^C^ in α-Synuclein Binding

It has been described how the residues of the CC of PrP^C^ are involved in binding β-amyloid with PrP^C^ [53]. Hence, we aimed to determine whether this domain also participates in α-synuclein binding (Fig. 6). After cloning, the expression of all PrP^C^ variants in HEK293 cells were tested by western blotting (Supplementary Fig. 5). The endogenous level of *Prnp* expression in HEK293 was low (Fig. 5a), and all PrP^C^-modified proteins were detectable (Supplementary Fig. 5). However, expression of ΔF35 was markedly lower than that of the rest of the deleted forms, representing less than 50% of the expression of full-length PrP^C^ (Supplementary Fig. 5). At this point, we considered the fact that some of these constructs are able to induce cell death when overexpressed in cell lines [54, 1]. Results indicate that only ΔF35 increased caspase 3 activity in transfected cells in contrast to other PrP^C^ constructs (relative fluorescence units (RFU) ΔF35/pcDNA = 2.15 ± 0.38; ΔCC/pcDNA = 0.92 ± 0.27; ΔHR/pcDNA = 1.11 ± 0.21; PrP^C^/pcDNA = 1.14 ± 0.09; mean ± SEM) (Supplementary Fig. 5). After these results, we ignored ΔF35 in the next experiments and focused on the central domain (ΔCC and ΔHR) of PrP^C^ (Figs. 5 and 6). In the experiments, we reduced the amount of the cDNA to half during cell transfection to ensure clear immunocytochemical detection of transfected cells. After transfection and immunocytochemistry, only 5.83% (22 of 377, *n* = 3) of the ΔCC-labelled cells were α-synuclein-positive (Fig. 6d–g) in contrast to ΔHR/α-synuclein (96.49% (110 of 114, *n* = 3)) (Fig. 6h–k) and PrP^C^/α-synuclein (91.08% (286 of 314 cells, *n* = 3) (Fig. 5c–h and Fig. 6b, c), indicating the participation of the CC domain of PrP^C^ in α-synuclein binding to PrP^C^-transfected HEK293 cells.

## Discussion

In this study, we determined that α-synuclein fibrils can be transported to different brain regions of wild-type mice after injection in the postcommissural striatum. Our results reinforce the notion that the spreading of p-α-synuclein pathology does not occur by diffusion or non-specific transport [45]. The transport observed in our experiments was similar to that reported in other studies using wild-type mice [29, 28], with relevant p-α-synuclein deposits in the striatum, substantia nigra, amygdala and neocortex. The anatomical connections between the striatum, substantia nigra, amygdala and neocortex are well described in the literature (e.g. [55]) and support the observed transport of α-synuclein. Although it has been reported to exist anterograde and retrograde transport of α-synuclein (see above), in our experiments, transported α-synuclein seemed to be more often transported retrogradely in the brain parenchyma, as suggested in other works [29, 28]. Our results also demonstrate for the first time that p-α-synuclein pathology after injection could spread to different brain regions in the absence of PrP^C^. Thus, *Prnp* expression is not mandatory for α-synuclein transport in the mouse brain, although increased levels of α-synuclein transport can be seen in wild-type and overexpressing mice. In fact, it has been reported that the absence of PrP^C^ does not modify the appearance or temporal evolution of p-α-synuclein deposits in transgenic mice overexpressing human α-synuclein driven by a platelet-derived growth factor-β promoter [56]. Furthermore, changes on *Prnp* dosage do not alter α-synuclein expression in adult mice (not shown). Although data using additional models of synucleinopathies (e.g. A53T mice) have not been published, the present results are in line with these observations [56]. However, we also determined an increased number of motor pyramidal neurons displaying LBL aggregates in *Prnp*-overexpressing mice which suggest that, as also reported for β-amyloid, PrP^C^ might participate in the cellular binding of α-synuclein and its expansion.

The interaction of several amyloids to PrP^C^ is well characterized [14, 6, 4, 15–17]. We determined, using two different *Prnp* constructs, that their overexpression can enhance the binding of α-synuclein in HEK293 cells with low endogenous capability of α-synuclein binding (see also [48] for details). Several studies have reported the interaction of endogenous peptides of the amyloid family with the plasma membrane [57–61]. In fact, the interaction of Aβ, human or rat amylin, PrP_(106–126)_ or α-synuclein fibrils with plasma membrane has been well described [57–60, 62], and several putative interactions with plasma membrane proteins have been described as well (e.g. APP [59] or GRP78 [51]). To this concern, a recent study of Mao and coworkers [48] reviewed in [63] points to the lymphocyte-activation gene 3 (LAG3/CD223) as the neuronal receptor of α-synuclein. LAG3 showed increased binding properties to α-synuclein protofibrils as compared to neurexin 1β, and APLP1 in SH-SY5Y-overexpressing cells [48]. mRNA levels of LAG3 do not change between *Prnp* genotypes (GEO database ref. GSE16223). However, and since a functional interaction between LAG3 with PrP^C^ cannot be ruled out, we hypothesise that PrP^C^ as well as other proteins (e.g. GRP78) may cooperate with LAG3 in neuronal α-synuclein transport. This is also relevant if considered that the absence of LAG3 does not fully impair α-synuclein protofibril transport in vitro and in vivo [48].

Although not described for other fibrillar peptides, PrP^C^ has been reported as a receptor for Aβ (see above). The present study indicates that PrP^C^ might contribute to enhanced binding of α-synuclein fibrils to the plasma membrane, in line with other studies, reporting an increased level of binding between Aβ and PrP^C^ during ageing in several mouse models of Alzheimer’s disease [15]. In addition, our data suggest that the CC domain actively participates in α-synuclein binding. Residues located in the CC domain (aa 90–110 or aa 91–115) have been involved in binding with β-amyloid [64, 53] in cooperation with the N-terminal residues 23–58 [53]. As these PrP^C^ domains have been revealed as putative pharmacological targets for Alzheimer’s disease [65–67], our data might also enhance the eligibility of PrP^C^ as a putative target to modulate α-synuclein expansion.

## Electronic Supplementary Material

Supplementary Fig. 1. **a-b** Electron microscopy photomicrograph illustrating recombinant mouse α-synuclein fibers in aggregated stage (**a**) and after sonication procedure (**b**). **c** Western blot of sonicated α-synuclein fibers. Note the appearance of the ≈ 17 kDa band as well as the ≈ 35 kDa band typical of non-monomeric α-synuclein. Similar band pattern was observed in the case of human α-synuclein fibrils **d-e** Low power photomicrographs illustrating p-α-synuclein staining in sham-operated (**d**) and mouse α-synuclein-operated (**e**) *Prnp* ^*+/+*^ mice. p129S/81A p-α-synuclein labelling can be seen in parietal and cingular neocortex, hippocampus, mamillothalamic tract, fornix, globus pallidus, caudate putamen and white matter. However, after injection, p-α-synuclein deposits as LBL or LNL aggregates (*arrows* in **e**) can clearly be seen over the background using the p129S/81A antibody. **f-g** High power photomicrographs illustrating p-α-synuclein LBL after mouse fibril injection in neocortex (**f**) or amygdala (**g**). Abbreviations: *A*, amygdala; *CP*, caudate putamen; *EC*, entorhinal cortex; *GP*, globus pallidus; *H*, hippocampus; *T*, thalamus. *Scale bar*: *d* = 500 μm pertains to **e**; *f* = 25 μm pertains to **g**. (JPEG 8544 kb)

Supplementary Fig. 2. Analysis of mouse α-synuclein transport using microfluidic devices. **a-b** Examples of Fluo4-AM labelling (FITC optics) of microfluidic devices after 7 DIV. Note the difference in the number of labelled axons between (**a**) and (**b**) in the microchannels. **c** Example of western blotting of α-synuclein in a device without relevant interconnection reservoirs (a case). Two different exposures (10 seg and 4 min) are shown in the panel. Note the presence of a very pale band of α-synuclein in the B cell extract (*asterisk*) only detectable after 4 min of exposure. In these cultures, no endogenous α-synuclein labelling was observed, demonstrating detected α-synuclein in B derived from interneuronal transport of exogenous α-synuclein protofibrils as also demonstrated in α-synuclein (Fig. 4a-d) p-α-synuclein staining (Fig. 3e-h, Supplementary Fig. 3). Anti-β-tubulin was used for protein characterization. *Scale bar*: *a* = 400 μm pertains to **b**. (JPEG 3042 kb)

Supplementary Fig. 3. Analysis of human α-synuclein fibril transport using microfluidic devices. **a** 2D representation of the two PDMS devices used in the present study. **b-g** Primary cortical cultures of *Prnp* ^+/+^ were maintained in the devices for 5-7 days. Then human recombinant α-synuclein was added to A reservoir (*asterisk*) (**b**,**d**,**f**), neuronal presence of p-α-synuclein in A was analysed (**c**), and their transport to B reservoir was analysed with p-α-synuclein immunocytochemistry (**d-g**). **b-c** Examples of double-labelled neurons (TUJ1/p-α-synuclein) in A reservoir (indicated with camera icon) showing p-α-synuclein labelling as LBL (*arrows* in **b** and **c**). **d-g** Examples of p-α-synuclein-labelled neurons and axons (TUJ1-positive, *arrows*) in B reservoir (indicated with camera icon). *Scale bar*: b and f = 40 μm pertains to **c**-**e** and **g**, respectively. (JPEG 8502 kb)

Supplementary Fig. 4. Overexposed (15 min) uncropped films showing the absence of α–synuclein in cultured media of B in contrast to A reservoir, indicating the absence of fluidic flux between reservoirs illustrated in Fig. 3i. (JPEG 2392 kb)

Supplementary Fig. 5. **a** Western blot illustrating the overexpression of the different PrP^C^ constructs (ΔF35, ΔHR, ΔCC, and PrP^C^) in HEK293 cells. The cellular distribution of the constructs in the plasma membrane can be seen in [54] **b** Histogram illustrating the activation of caspase 3 by the different PrP^C^ constructs (including ΔCD and ΔCR constructs). Data represent the mean ± S.E.M. of three different experiments. *** *P* < 0.01, ANOVA Bonferroni post hoc test. (JPEG 1479 kb)
