# Supplementary figures and images for: Involvement of Cellular Prion Protein in α-Synuclein Transport in Neurons
Source: Mol Neurobiol. 2017 Feb 22;55(3):1847–60. doi: 10.1007/s12035-017-0451-4 (PMC5840251; doi:10.1007/s12035-017-0451-4)

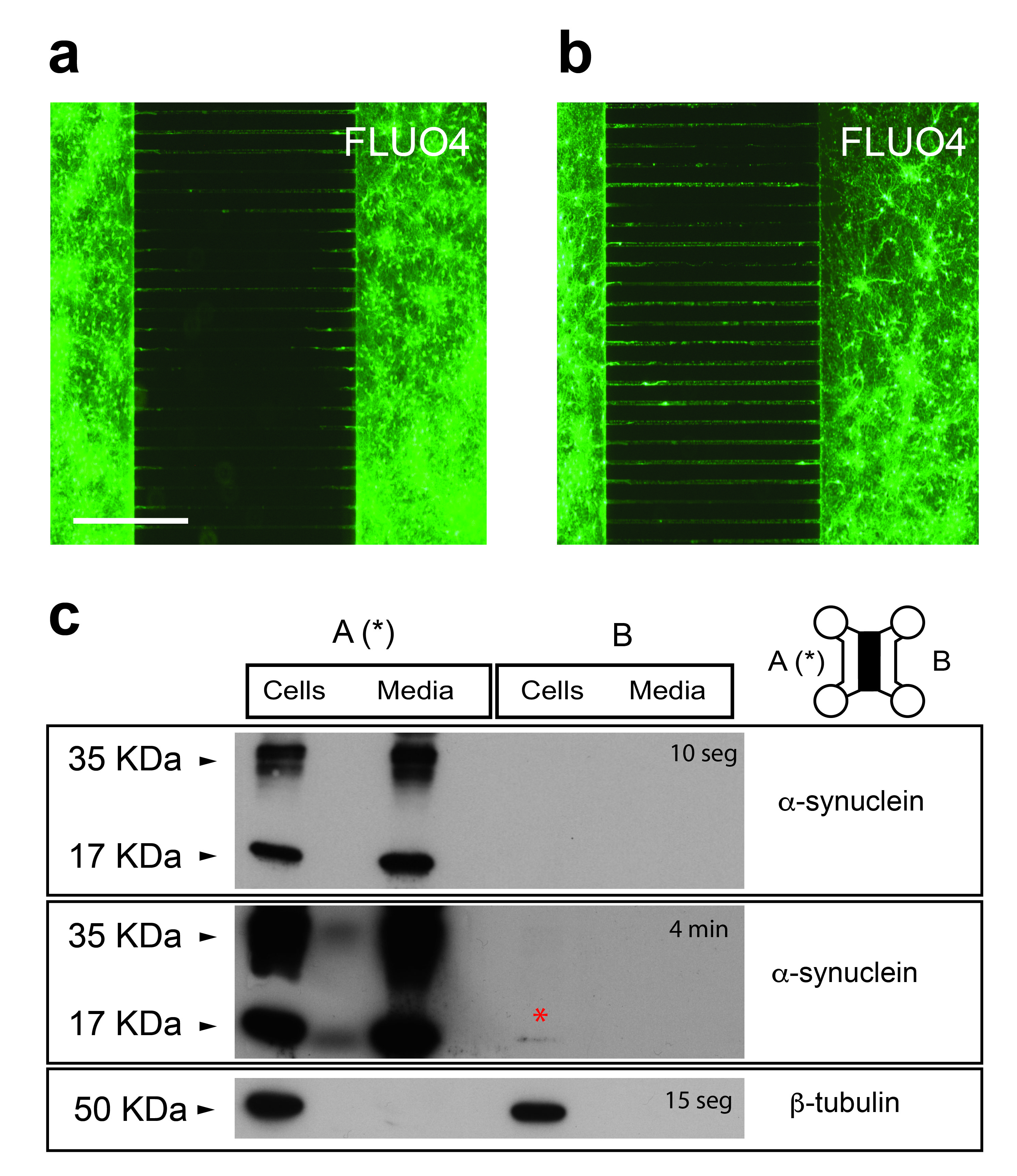

Supplement: Supplementary file 2 — Analysis of mouse α-synuclein transport using microfluidic devices. a-b Examples of Fluo4-AM labelling (FITC optics) of microfluidic devices after 7 DIV. Note the difference in the number of labelled axons between (a) and (b) in the microchannels. c Example of western blotting of α-synuclein in a device without relevant interconnection reservoirs (a case). Two different exposures (10 seg and 4 min) are shown in the panel. Note the presence of a very pale band of α-synuclein in the B cell extract (asterisk) only detectable after 4 min of exposure. In these cultures, no endogenous α-synuclein labelling was observed, demonstrating detected α-synuclein in B derived from interneuronal transport of exogenous α-synuclein protofibrils as also demonstrated in α-synuclein (Fig. 4a-d) p-α-synuclein staining (Fig. 3e-h, Supplementary Fig. 3). Anti-β-tubulin was used for protein characterization. Scale bar: a = 400 μm pertains to b. (JPEG 3042 kb) [file 12035_2017_451_Fig8_ESM.jpg]

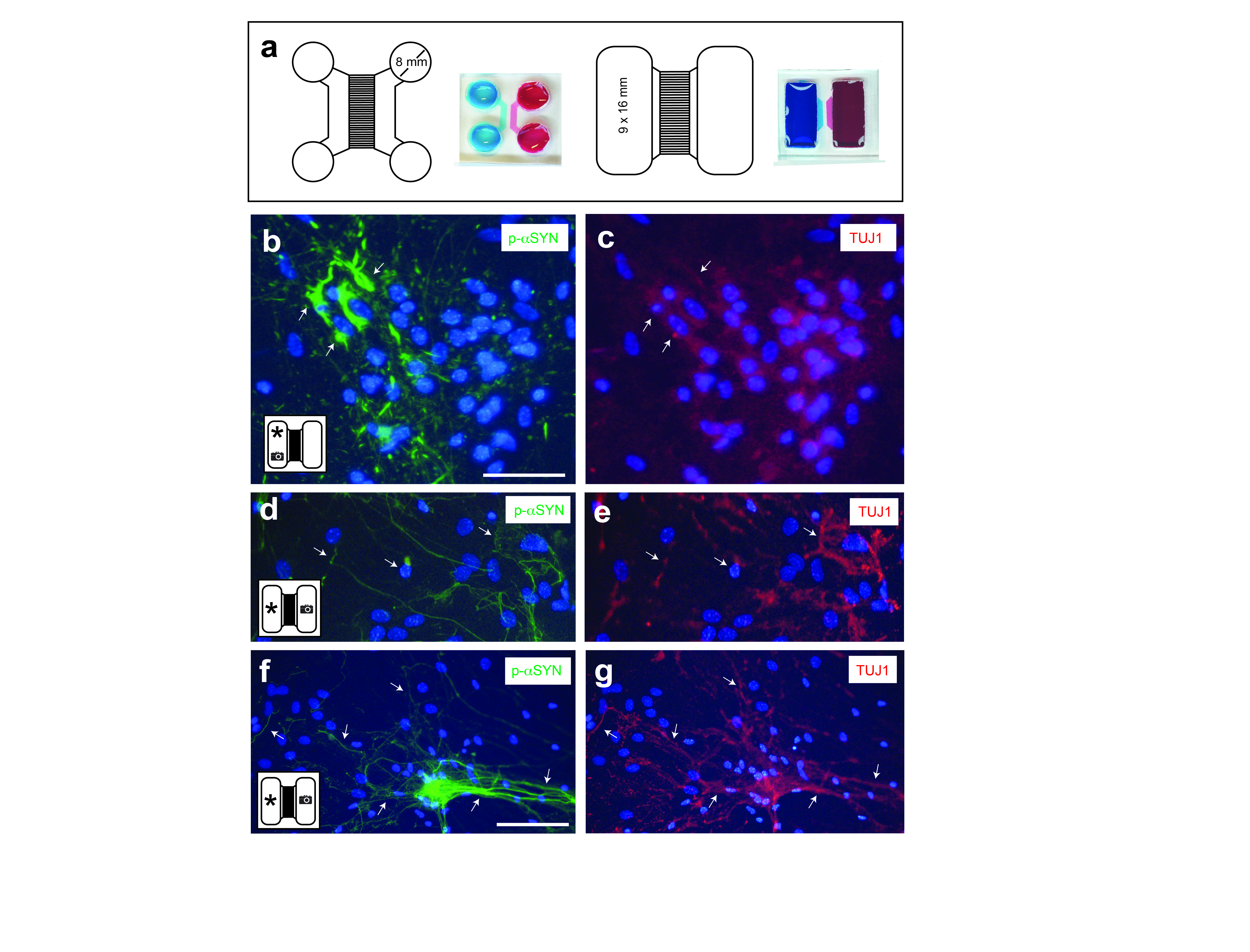

Supplement: Supplementary file 3 — Analysis of human α-synuclein fibril transport using microfluidic devices. a 2D representation of the two PDMS devices used in the present study. b-g Primary cortical cultures of Prnp +/+ were maintained in the devices for 5-7 days. Then human recombinant α-synuclein was added to A reservoir (asterisk) (b,d,f), neuronal presence of p-α-synuclein in A was analysed (c), and their transport to B reservoir was analysed with p-α-synuclein immunocytochemistry (d-g). b-c Examples of double-labelled neurons (TUJ1/p-α-synuclein) in A reservoir (indicated with camera icon) showing p-α-synuclein labelling as LBL (arrows in b and c). d-g Examples of p-α-synuclein-labelled neurons and axons (TUJ1-positive, arrows) in B reservoir (indicated with camera icon). Scale bar: b and f = 40 μm pertains to c-e and g, respectively. (JPEG 8502 kb) [file 12035_2017_451_Fig9_ESM.jpg]

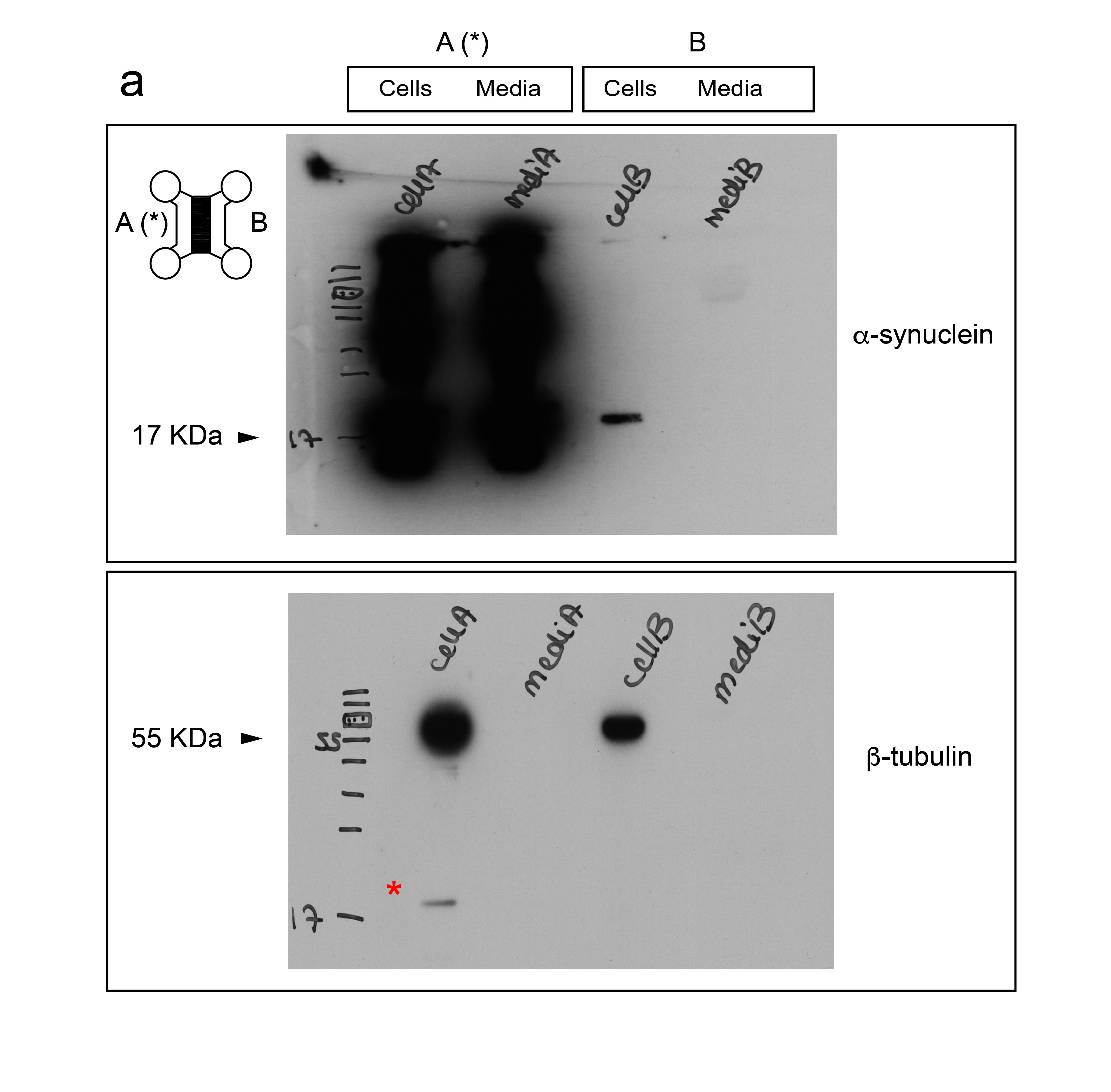

Supplement: Supplementary file 4 — Overexposed (15 min) uncropped films showing the absence of α–synuclein in cultured media of B in contrast to A reservoir, indicating the absence of fluidic flux between reservoirs illustrated in Fig. 3i. (JPEG 2392 kb) [file 12035_2017_451_Fig10_ESM.jpg]

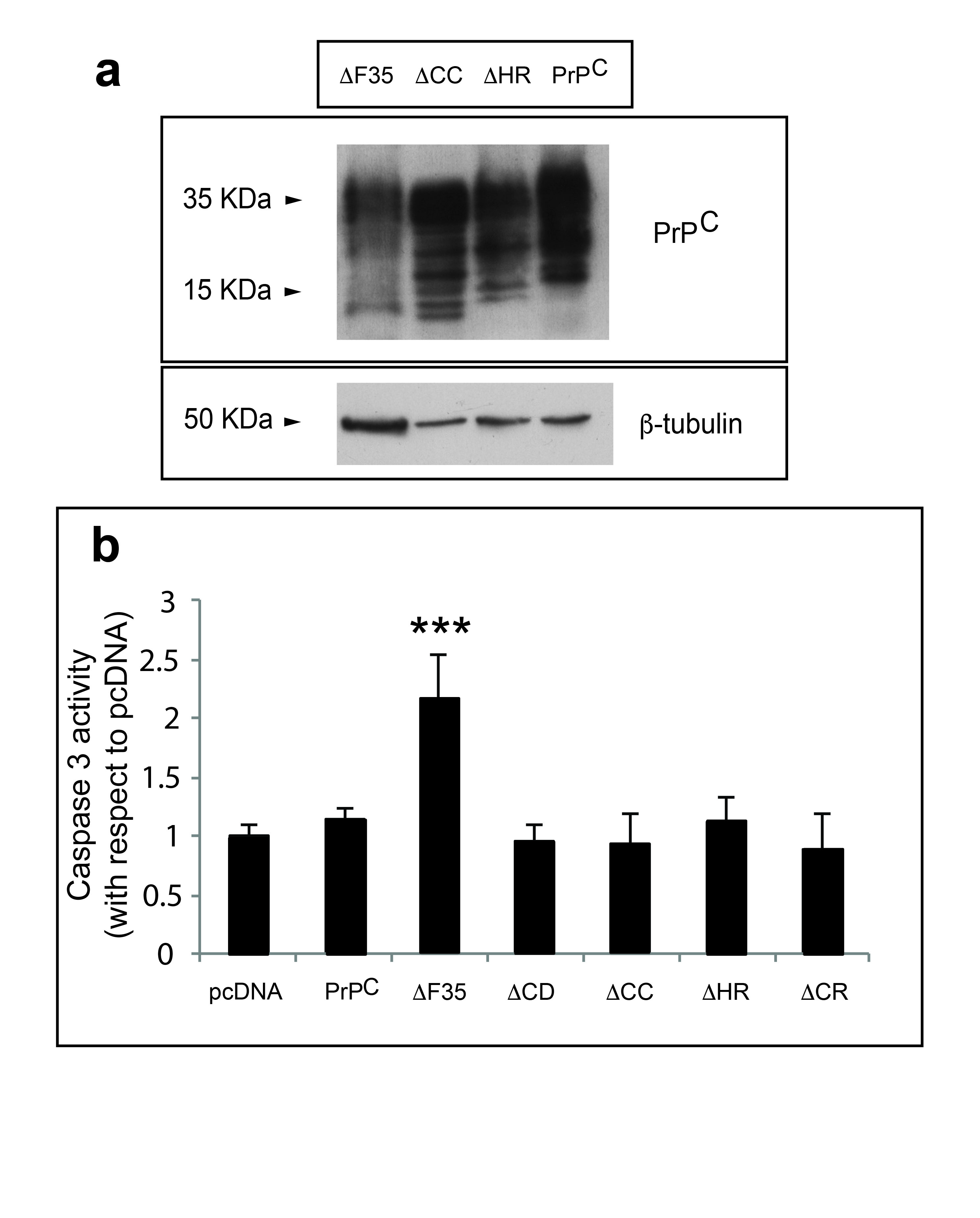

Supplement: Supplementary file 5 — a Western blot illustrating the overexpression of the different PrPC constructs (ΔF35, ΔHR, ΔCC, and PrPC) in HEK293 cells. The cellular distribution of the constructs in the plasma membrane can be seen in [54] b Histogram illustrating the activation of caspase 3 by the different PrPC constructs (including ΔCD and ΔCR constructs). Data represent the mean ± S.E.M. of three different experiments. *** P < 0.01, ANOVA Bonferroni post hoc test. (JPEG 1479 kb) [file 12035_2017_451_Fig11_ESM.jpg]
